# A Novel Murine Model of Hemophagocytic Lymphohistiocytosis‐Like Inflammation in ZNFX1 Deficiency

**DOI:** 10.1002/eji.70141

**Published:** 2026-02-04

**Authors:** Diana Tintor, Samantha Milanesi, Tommaso Marchetti, Tiziana Lorenzini, Severin Walser, Junyi Chen, Julius Köppen, Achim Weber, Ola Sabet, Jana Pachlopnik Schmid

**Affiliations:** ^1^ Division of Immunology and University Children's Hospital Research Center University Children's Hospital Zurich Zurich Switzerland; ^2^ Pediatric Immunology Faculty of Medicine University of Zurich Zurich Switzerland; ^3^ Department of Pathology and Molecular Pathology University Hospital Zurich and University of Zurich Zurich Switzerland; ^4^ Institute of Molecular Cancer Research (IMCR) University of Zurich Zurich Switzerland; ^5^ Children's Cancer Hospital Egypt 57357 Cairo Egypt

**Keywords:** Hemophagocytic lymphohistiocytosis (HLH), Macrophage activation, Mouse model, Viral infection, ZNFX1 deficiency

## Abstract

Hemophagocytic lymphohistiocytosis (HLH) is a severe inflammatory syndrome characterized by persistent activation of lymphocytes and macrophages. Recently, deleterious autosomal recessive mutations in *ZNFX1* were reported to predispose pediatric patients to HLH‐like disease upon viral trigger. The objective of this study was to assess the suitability of Znfx1‐mutant (Znfx1^mut^) mice infected with lymphocytic choriomeningitis virus (LCMV) as a model of HLH‐like inflammation observed in patients. Following LCMV infection, Znfx1^mut^ mice were monitored for pathophysiological signs of HLH, and their cells were immunophenotyped. Furthermore, functional assays were performed in vitro on T cells and bone marrow‐derived macrophages (BMDMs) to assess the cells’ response to stimuli. Our experiments highlighted several hallmark features of HLH‐like inflammation in Znfx1^mut^ mice. Immunophenotyping revealed more pronounced T cell expansion and type‐1 helper (Th1) polarization in LCMV‐infected Znfx1^mut^ mice. Znfx1^mut^ macrophages infiltrated the liver to a greater extent upon infection and produced greater levels of cytokines in vitro in the absence of stimulation, suggesting that these cells have a major role in driving inflammation. This novel murine model of HLH‐like inflammation mirrors key aspects of the immune dysregulation observed in patients, providing a valuable tool for studying disease mechanisms in ZNFX1 deficiency.

## Graphical abstract

LCMV‐infected Znfx1‐deficient mice develop hemophagocytic lymphohistiocytosis (HLH)‐like immune dysregulation, mirroring key aspects of the immunopathology observed in ZNFX1‐deficient patients.

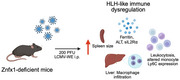

**Abbreviations**
ALT: alanine aminotransferase
BMDM: bone marrow‐derived macrophage
EM: effector memory
HLH: hemophagocytic lymphohistiocytosis
IFN: interferon
IL: interleukin
LCMV: lymphocytic choriomeningitis virus
M‐CSF: macrophage colony‐stimulating factor
p.i.: post infection
PFU: plaque‐forming units
sIL2R⍺: soluble IL2 receptor ⍺
ssRNA: single‐stranded RNA
Th1: type‐1 helper
TNF‐⍺: tumor necrosis factor alpha
WBC: white blood cell
Znfx1^mut^ mice: zinc finger NFX1‐type containing 1 knock‐out mutant mice

## 1 Introduction

Hemophagocytic lymphohistiocytosis (HLH) is a severe inflammatory syndrome with multiorgan involvement. Primary HLH results from monogenic defects in the immune system that predispose patients to immune dysregulation [1, 2, 3]. Viral infections are one of the key triggers of disease onset, initiating a pro‐inflammatory signaling cascade that the immune system then fails to resolve [4]. As HLH does not have any pathognomonic signs, the diagnosis depends on the presence of clinical criteria. These criteria include fever, splenomegaly, cytopenia, an elevated triglyceride level, a low fibrinogen level, hemophagocytosis, hyperferritinemia, and an elevated soluble IL‐2 receptor ⍺ (sIL2R⍺) level [5].

The best‐characterized genetic causes of primary HLH impair lymphocyte cytotoxicity [1, 2]. In these cases, defective elimination of infected target cells results in prolonged antigen presentation and sustained activation of interferon (IFN)‐γ‐producing T cells, fueling a pathogenic loop of macrophage activation and excessive cytokine secretion [6, 7]. However, HLH can also result from mutations in genes involved in negative regulation of macrophage activation or cytokine control, highlighting that multiple immune pathways can converge on HLH‐like pathology [2, 8].

We and others recently described a novel inborn error of immunity caused by biallelic deleterious mutations in zinc finger NFX1‐type containing 1 (*ZNFX1*) [9, 10, 11]. Patients with ZNFX1 deficiency mostly presented within the first few years of life, and a subset developed HLH or HLH‐like syndromes following viral infection. Disease onset was most frequently associated with single‐stranded RNA (ssRNA) viruses, and episodes occurring after exposure to vaccine strains have indicated heightened susceptibility to infection [9, 10, 11]. Additional clinical features include leukocytosis during confirmed or suspected viral infections in a substantial subset of patients (8 of 15 in our cohort) [9], a finding that is unusual in classical HLH, and episodes of liver failure, reflecting severe systemic inflammation [9, 10].

ZNFX1 is an interferon‐stimulated gene (ISG) with an RNA helicase domain and E3 ligase activity [12, 13, 14]. Upon viral infection, ZNFX1 acts as an early RNA sensor binding directly to viral RNA and promoting a type I IFN response [12, 13, 14]. Recently, it has been shown that binding to ssRNA induces ZNFX1‐dependent ubiquitination and the formation of ubiquitinated ZNFX1/RNA condensates, which are crucial for cell survival during an IFN response [13]. In addition, the interaction of ZNFX1 with the specific host mRNA species Prkaa2 has been shown to stabilize Prkaa2, which is necessary for effective autophagy‐dependent immunity against Mycobacterium Tuberculosis [15]. Consistent with this, ZNFX1‐deficient patient cells show prolonged ISG mRNA stability when stimulated with viral mimics [9]. This suggests that ZNFX1 is essential for regulating RNA‐based innate immune responses and preventing excessive or dysregulated IFN signaling.

Mouse models have been instrumental in elucidating HLH pathogenesis—especially the molecular impact of monogenic cytotoxic defects—which has improved both the diagnosis and the treatment of HLH [16, 17, 18, 19, 20]. In this study, we infected Znfx1^mut^ mice with lymphocytic choriomeningitis virus (LCMV), a noncytopathic ssRNA virus capable of infecting a broad range of cells and widely used to model infection‐triggered immunopathology [21, 22, 23]. The objectives of this study were to establish a virally induced murine model of ZNFX1 deficiency and to evaluate HLH‐related features previously reported in patients. To the best of our knowledge, this study is the first to examine HLH criteria in Znfx1‐deficient mice.

## 2 Results

### 2.1 Characterization of Znfx1^mut^ Mice Infected with LCMV as a Model of HLH‐Like Immune Dysregulation

**FIGURE 1:**
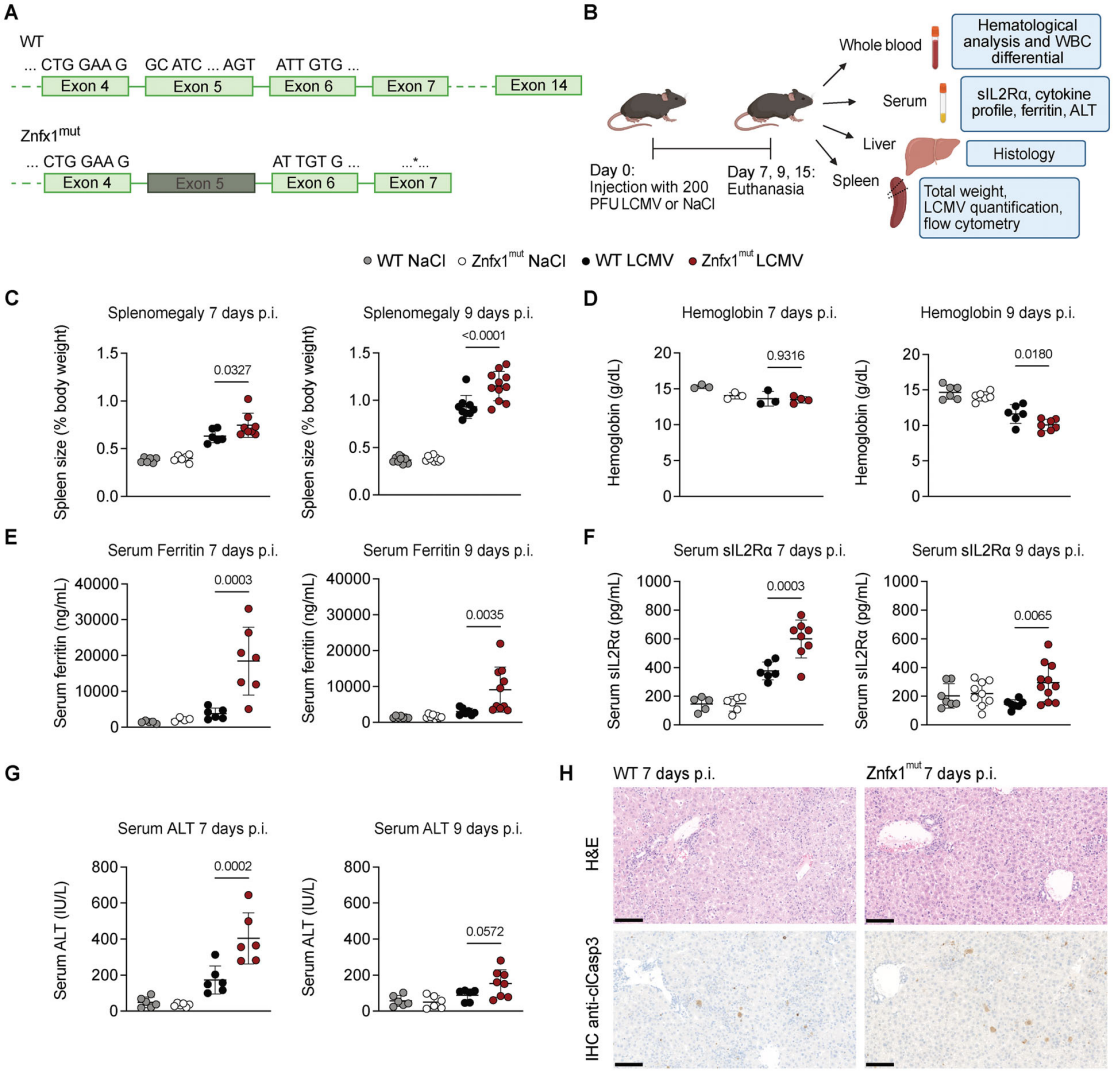
LCMV‐infected Znfx1^mut^ mice develop transient, HLH‐like inflammation that mimics clinical aspects of patients with ZNFX1 deficiency. (A) Schematic representation of the *Znfx1* gene in wildtype (WT) and the CRISPR‐generated *Znfx1* knock‐out mutant (Znfx1^mut^) mice. * indicates an early stop codon in exon 7. (B) Schematic representation of the experimental set‐up and the downstream sample analysis. (C) Spleen size as a percentage of body weight 7 days (left panel) and 9 days (right panel) post infection (p.i.) with 200 PFU of LCMV strain WE. (D) Hemoglobin levels in whole blood 7 days (left panel) and 9 days (right panel) p.i. with 200 PFU of LCMV strain WE. (E) Serum ferritin levels measured by ELISA 7 days (left panel) and 9 days (right panel) p.i. with 200 PFU of LCMV strain WE. (F) Serum soluble IL2Rα levels measured by ELISA 7 days (left panel) and 9 days (right panel) p.i. with 200 PFU of LCMV strain WE. (G) Serum alanine aminotransferase (ALT) levels measured by ELISA 7 days (left panel) and 9 days (right panel) p.i. with 200 PFU of LCMV strain WE. (H) Liver sections taken 7 days p.i. with 200 PFU of LCMV strain WE. Upper panels: hematoxylin and eosin (H&E) stain. Lower panels: anti‐cleaved caspase 3 (clCasp3) immunohistochemical (IHC) stain. 20× magnification, scale bar: 100 µM. For all plots, the data were analyzed in a one‐way analysis of variance with Šidák's correction for multiple comparisons. The graphs display the mean and the standard deviation. Each dot represents an individual mouse, and (except for hemoglobin measurements 7 days p.i.), all plots show the data from at least two independent experiments. *p*‐values are indicated above the plots.

To investigate HLH‐like inflammation in a murine model of ZNFX1 deficiency, we opted to study the inflammatory response upon viral infection. CRISPR‐generated *Znfx1* knock‐out mutant mice (Znfx1^mut^), lacking exon 5 of the *Znfx1* gene, were commercially available. The deletion in the helicase domain disrupts the reading frame and creates a premature stop codon in exon 7, as confirmed by Sanger sequencing (Figure 1A; Figure S1A). Znfx1^mut^ mice were infected with LCMV strain WE and euthanized on days 7, 9, or 15 post infection (Figure 1B).

Both wild‐type (WT) and Znfx1^mut^ mice showed comparable weight loss until day 7 post infection, with full recovery by day 15, consistent with successful resolution of infection (Figure S1B). RT‐qPCR analysis of spleen tissue showed similar levels of LCMV RNA in WT and Znfx1^mut^ mice at day 7, followed by a marked decline by day 9 post infection (Figure S1C). By day 15, viral RNA was undetectable in both groups, indicating effective viral clearance.

We next assessed features associated with HLH to determine if infected Znfx1^mut^ mice recapitulate clinical findings observed in patients with ZNFX1 deficiency. LCMV‐infected Znfx1^mut^ mice developed mild splenomegaly by day 7, which progressed by day 9 post infection (Figure 1C). Consistent with HLH‐associated hematologic abnormalities, Znfx1^mut^ mice exhibited significantly lower hemoglobin levels at day 9 (Figure 1D). Serum ferritin, sIL2R⍺, and alanine aminotransferase (ALT) were all significantly elevated at day 7 in infected Znfx1^mut^ mice compared with WT controls, with partial normalization by day 9 (Figure 1E–G).

Histological examination of liver tissue revealed mild architectural disarray, portal and lobular inflammation, and disseminated hepatocyte apoptosis in both genotypes, with slightly more pronounced pathology in Znfx1^mut^ mice (Figure 1H). In line with the ALT elevation and histologic findings, immunohistochemical staining showed increased numbers of cleaved caspase‐3‐positive hepatocytes in Znfx1^mut^ livers relative to WT controls at this time point (Figure 1H).

No differences were observed between infected WT and Znfx1^mut^ mice with respect to platelet counts, neutrophil counts, or serum cytokine levels (Figure S1D–F). IFN‐γ and, to a lesser extent, tumor necrosis factor‐alpha (TNF‐⍺) were the dominant circulating cytokines at day 7 in both genotypes, decreasing by day 9 post infection (Figure S1F). By day 15, all measured parameters had returned to baseline, indicating that the inflammatory phenotype was transient.

In summary, LCMV‐infected Znfx1^mut^ mice developed transient signs of HLH‐like inflammation—including splenomegaly, anemia, hyperferritinemia, elevated sIL2R⍺, and liver injury—mirroring aspects of the immune dysregulation observed in ZNFX1‐deficient patients following viral exposure.

### 2.2 Enhanced Th1 Polarisation in Znfx1^mut^ Mice Following LCMV Infection

**FIGURE 2:**
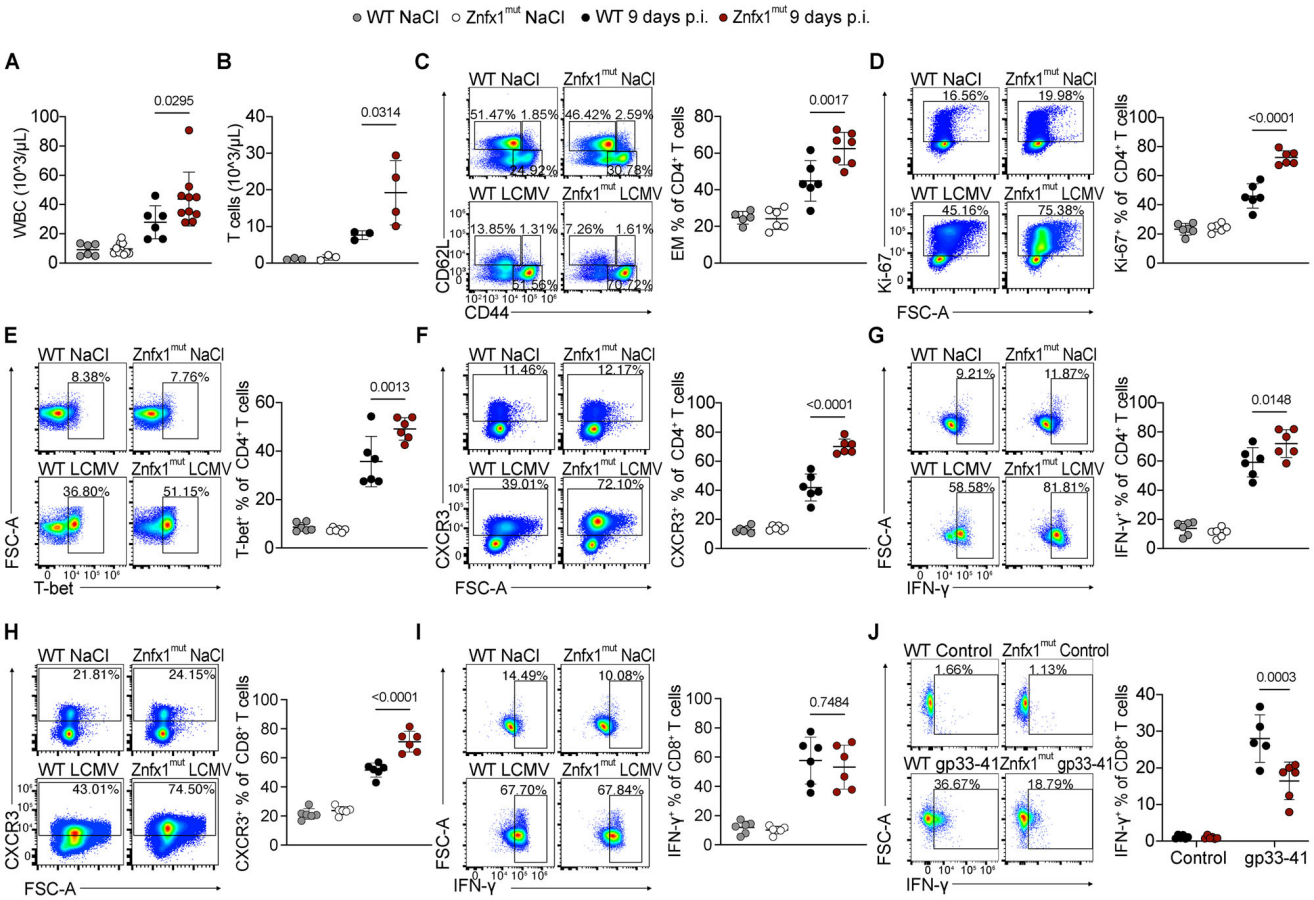
LCMV‐infected Znfx1^mut^ mice show T cell expansion and more pronounced Th1 polarisation. (A) The white blood cell (WBC) count in whole blood. (B) The T cell count in whole blood. (C) Flow cytometry plot of CD62L and CD44 expression on CD4^+^ T cells in the spleen. EM: effector memory. (D) Flow cytometry plot of Ki‐67 expression on CD4^+^ T cells in the spleen. (E) Flow cytometry plot of T‐bet expression in CD4^+^ T cells in the spleen. (F) Flow cytometry plot of CXCR3 expression on CD4^+^ T cells in the spleen. (G) Flow cytometry plot of IFN‐γ expression in CD4^+^ T cells taken from the spleen after in vitro stimulation with 0.08 µM phorbol 12‐myristate 13‐acetate, 1.34 µM ionomycin, and brefeldin A for 5 h. (H) Flow cytometry plot of CXCR3 expression on CD8^+^ T cells in the spleen. (I) Flow cytometry plot of IFN‐γ expression in CD8^+^ T cells taken from the spleen after in vitro stimulation with 0.08 µM phorbol 12‐myristate 13‐acetate, 1.34 µM ionomycin, and brefeldin A for 5 h. (J) Flow cytometry plot of IFN‐γ expression in CD8^+^ T cells after in vitro stimulation with 10 µM gp33‐41 and brefeldin A for 5 h. All plots show cells from mice mock‐infected with NaCl or 9 days post infection (p.i.) with 200 PFU of LCMV strain WE. The data were analyzed in a one‐way analysis of variance with Šidák's correction for multiple comparisons. The graphs display the mean and the standard deviation. Each dot represents an individual mouse, and (except for the T cell count in blood) all plots show the data from at least two independent experiments. *p*‐values are indicated above the plots.

After establishing that Znfx1^mut^ mice develop signs of HLH upon viral infection, we used blood analyses and splenocyte immunophenotyping to characterize differences between infected Znfx1^mut^ and WT mice on the cellular level. At day 9 post infection, Znfx1^mut^ mice had a significantly higher white blood cell (WBC) count than the infected WT mice did (Figure 2A). In Znfx1^mut^ mice, the high WBC count was accompanied by a significantly elevated total T cell count in the blood (Figure 2B). We hypothesized that excessive T cell expansion is associated with differences in the T cell response.

In the spleen, CD8^+^ T cells were the predominant T cell subset 9 days post infection (Figure S2A). The effector memory (EM) CD4^+^, but not CD8^+^, T cell subset was significantly expanded in Znfx1^mut^ mice (Figure 2C; Figure S2B). Znfx1^mut^ mice also expressed significantly more Ki‐67 in CD4^+^, but not CD8^+^ T cells, indicating that the expansion of the EM CD4^+^ T cells was due to increased proliferation (Figure 2D; Figure S2C). Consistent with the more differentiated phenotype, Znfx1^mut^ CD4^+^ T cells had significantly higher expression of the transcription factor T‐bet and the inflammatory chemokine receptor CXC motif chemokine Receptor 3 (CXCR3) (Figure 2E,F). In vitro stimulation of T cells with phorbol 12‐myristate 13‐acetate (PMA) and ionomycin revealed significantly more IFN‐γ‐producing CD4^+^ T cells in Znfx1^mut^ mice compared with their WT counterparts (Figure 2G).

There were no differences in the expression of the early T cell activation markers OX40 and CD69, the differentiation markers CD27 and CD39, or the terminal differentiation markers death protein‐1/T‐cell immunoglobulin and mucin‐domain containing‐3 (PD‐1/Tim‐3) and PD‐1/Tox in either CD4^+^ or CD8^+^ T cells (Figure S2D–I). As CD4^+^ T cells expressing T‐bet, CXCR3, and IFN‐γ are considered type‐1 helper (Th1) polarized T cells [25, 26], these findings indicate that at day 9 post infection, Znfx1^mut^ CD4^+^ T cells display a more pronounced Th1 effector memory phenotype without increased signs of activation or terminal differentiation.

In line with the Th1‐skewed CD4^+^ T cell response, infected Znfx1^mut^ CD8^+^ T cells showed significantly higher upregulation of CXCR3 (Figure 2H). In vitro stimulation of the Znfx1^mut^ CD8^+^ T cells with PMA and Ionomycin revealed their propensity to produce IFN‐γ and Granzyme B, with expression levels similar to those observed for cells from WT mice (Figure 2I; Figure S2J). Znfx1^mut^ CD8^+^ T cells harvested 9 days post infection responded to in vitro restimulation with LCMV antigens, but produced significantly less IFN‐γ, while Znfx1^mut^ CD4^+^ T cells produced IFN‐γ levels similar to their WT counterparts (Figure 2J; Figure S2K). These results indicate that T cells taken from infected Znfx1^mut^ mice are capable of T cell receptor‐dependent recognition of LCMV antigens, followed by production of IFN‐γ, albeit to a lesser extent in the case of Znfx1^mut^ CD8^+^ T cells. In conclusion, Znfx1^mut^ mice showed more pronounced T cell expansion in the blood and a selective increase in splenic Th1‐skewed EM CD4^+^ T cells emerging in the context of infection.

### 2.3 Macrophage Infiltration of the Liver Is Increased in LCMV‐Infected Znfx1^mut^ Mice

**FIGURE 3:**
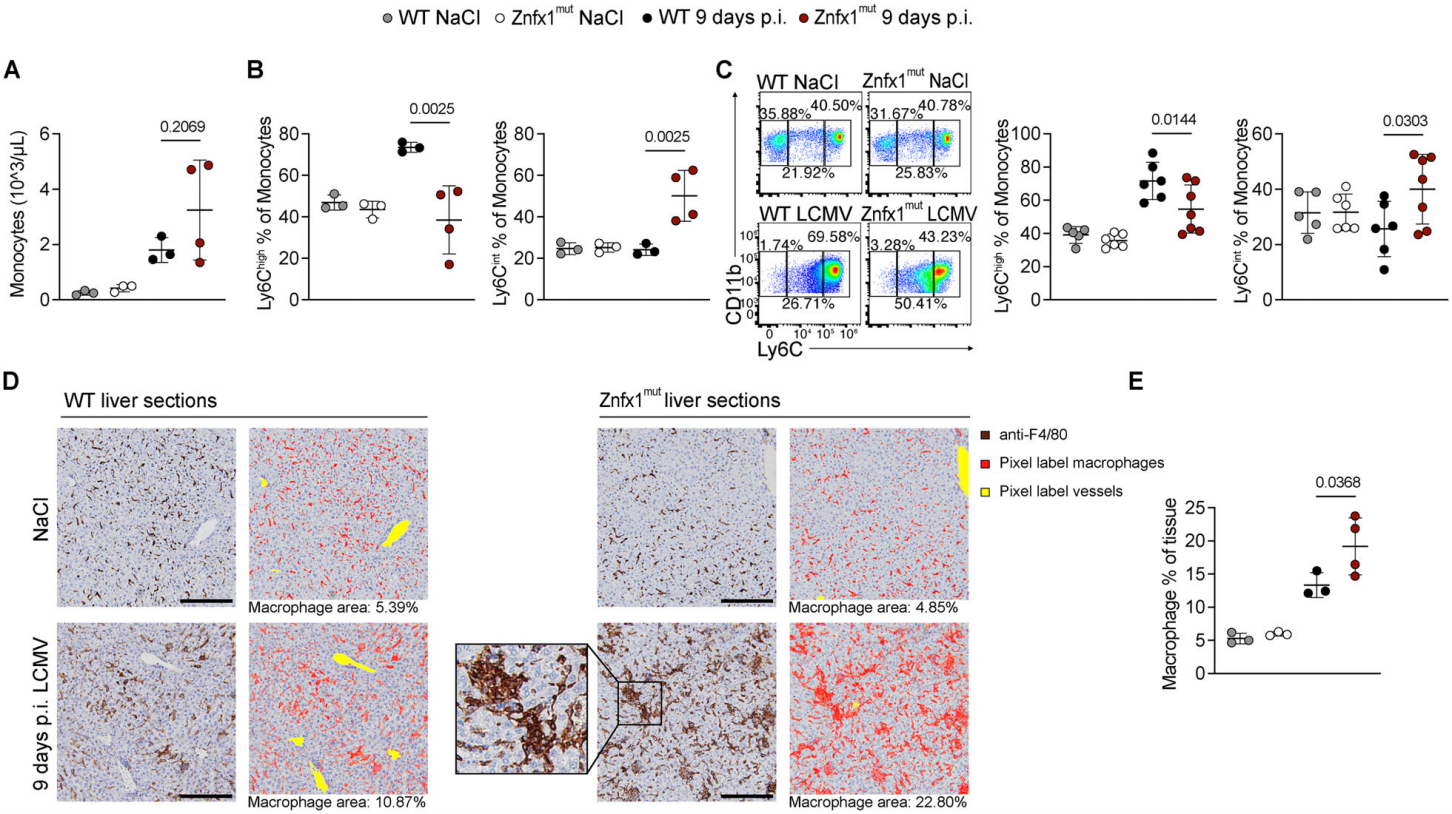
LCMV‐infected Znfx1^mut^ mice show altered Ly6C expression patterns in monocytes and a greater abundance of macrophages in the liver 9 days p.i. (A) The monocyte count in whole blood. (B) Ly6C^high^ (left panel) and Ly6C^intermediate^ (Ly6C^int^, right panel) monocytes in whole blood. (C) Flow cytometry plot of Ly6C^high^ (left panel) and Ly6C^int^ (right panel) monocytes in the spleen. (D) Liver sections immunohistochemically stained with anti‐F4/80, 20× magnification. Box: Hemophagocytosis. Scale bar: 100 µM. The artificial intelligence software Ilastik was trained to recognize pixels belonging to vessels and macrophages. (E) Quantification of F4/80^+^ macrophages in the liver. All plots show cells from mice mock‐infected with NaCl or 9 days post in vitro infection (p.i.) with 200 PFU of LCMV strain WE. The data were analyzed in a one‐way analysis of variance with Šidák's correction for multiple comparisons. The graphs display the mean and the standard deviation. Each dot represents an individual mouse. The data in A, B, D, and E are from one experiment, while 3C shows the data from two independent experiments. *p*‐values are indicated above the plots.

To assess the myeloid compartment of infected Znfx1^mut^ mice, we analyzed monocyte subsets and macrophage infiltration in LCMV‐infected Znfx1^mut^ mice. Although a few individual Znfx1^mut^ mice exhibited high monocyte counts at day 9 post infection, there was no significant monocytosis at the group level, as sometimes observed in ZNFX1‐deficient patients [10, 24] (Figure 3A).

Despite comparable total monocyte numbers, infected Znfx1^mut^ mice displayed a significant shift in monocyte subpopulations. Both blood and spleen monocytes showed a reduced proportion of lymphocyte antigen 6 family member C1 (Ly6C)^high^ monocytes, accompanied by an increased frequency of Ly6C^intermediate^ (Ly6C^int^) monocytes (Figure 3B,C). In contrast, the proportion of Ly6C^low^ monocytes was similar in Znfx1^mut^ and WT mice (Figure S3A,B). These findings indicate altered monocyte homeostasis or differentiation dynamics in Znfx1 deficiency during viral infection.

We next assessed macrophage populations in the liver. Immunohistochemical staining for the macrophage marker F4/80 revealed significantly increased macrophage abundance in infected Znfx1^mut^ mice, with occasional evidence of hemophagocytosis (Figure 3D,E). In contrast, staining for the Kupffer cell‐specific marker CLEC4F showed no difference between WT and Znfx1^mut^ mice (Figure S3C,D), indicating that the expanded F4/80^+^ population reflects infiltrating macrophages rather than an increase in resident Kupffer cells.

Together, these results demonstrate myeloid dysregulation in Znfx1^mut^ mice following LCMV infection, characterized by altered monocyte subset composition in the blood and spleen and enhanced macrophage infiltration into the liver.

### 2.4 In the Absence of Stimulation, Znfx1^mut^ Bone Marrow‐Derived Macrophages (BMDMs) Produce More TNF‐⍺, IL‐6, and IFN‐γ

Given the changes in monocyte dynamics observed in LCMV‐infected Znfx1^mut^ mice, we next sought to determine whether Znfx1 deficiency affects the intrinsic responsiveness of myeloid cells. Bone marrow monocytes were harvested for in vitro  analysis and subsequent differentiation into BMDMs. Before differentiation, Znfx1^mut^ monocytes displayed altered expression of the migration markers Ly6C and CX3CR1, whereas CD62L and CCR2 expression were comparable to WT cells (Figure S4A,B). At steady state or following 8 h stimulation with ssRNA40/Lyovec, monocytes of both genotypes did not differ in their production of TNF‐⍺ or Interleukin‐6 (IL‐6) (Figure S4C).

**FIGURE 4:**
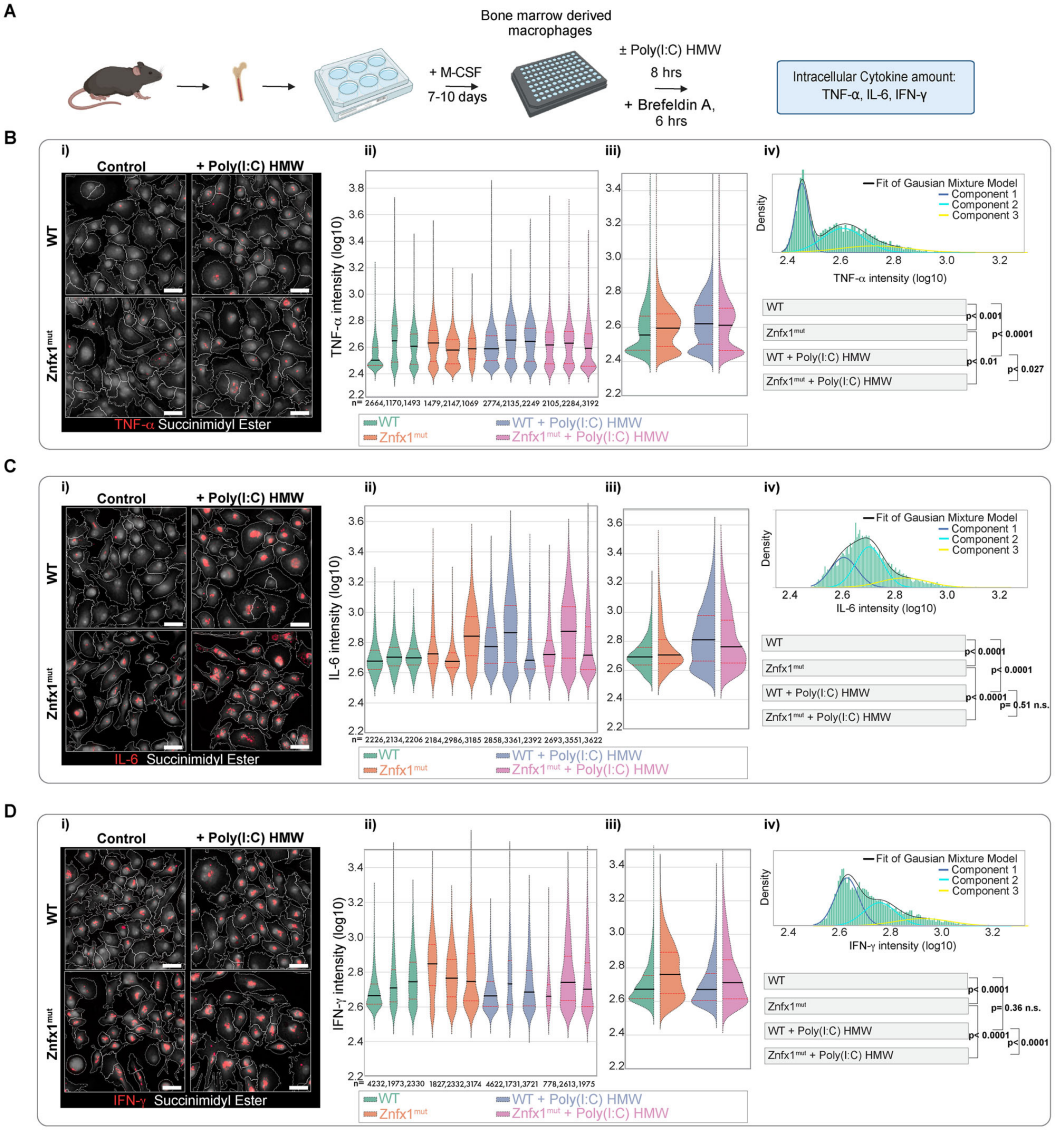
BMDMs from Znfx1^mut^ mice produce more pro‐inflammatory cytokines in an unstimulated state. (A) Schematic representation of the culture and stimulation of bone marrow‐derived macrophages (BMDMs). (B) TNF‐⍺ expression of BMDMs from WT and Znfx1^mut^ mice in an unstimulated control setting or after stimulation with 2 µg/mL Poly(I:C) HMW for 8 h, with brefeldin A for 6 h. (C) IL‐6 expression of BMDMs from WT and Znfx1^mut^ mice in an unstimulated control setting or after stimulation with 2 µg/mL Poly(I:C) HMW for 8 h, with brefeldin A for 6 h. (D) IFN‐γ expression of BMDM from WT and Znfx1^mut^ mice in an unstimulated control setting or after stimulation with 2 µg/mL Poly(I:C) HMW for 8 h, with brefeldin A for 6 h. Representative images are shown in panel (i). The measured log10 single‐cell intensity values are combined into violin plots for each mouse, and the number of measured cells per mouse is stated beneath the respective plot in panel (ii). The values for all the mice in the same group were grouped into violin plots in panel (iii). For the statistical analysis, a separate Gaussian mixture model (GMM) was fitted with the cell intensity values for each mouse. A weighted group mean and the covariance were calculated from the number of observations per mouse. Panel (iv) shows the resulting histogram for unstimulated WT cells with the combination of the weighted GMMs for the entire group (black line) and the three components of the fit (shown in dark blue, light blue, and yellow). The means were compared in a two‐tailed *t*‐test; *p*‐values are indicated. The data represent the results of one experiment, *n* = 3.

Differentiation with macrophage colony‐stimulating factor (M‐CSF) generated >95% F4/80^+^ CD11b^+^ macrophages for both Znfx1^mut^ and WT cells (Figure S4D). To assess macrophage cytokine production, BMDMs were analyzed by microscopy either under unstimulated conditions or after 8 h of stimulation with high‐molecular‐weight poly(I:C), a synthetic double‐stranded RNA mimic (Figure 4A).

Under unstimulated conditions, Znfx1^mut^ BMDMs exhibited significantly higher expression of TNF‐⍺, IL‐6, and IFN‐γ compared with WT BMDMs (Figure 4B–D). In all conditions, a subset of macrophages showed no detectable signal and were considered nonresponders. However, Znfx1^mut^ cultures contained a larger proportion of strongly responding cells than WT cultures.

Upon poly(I:C) stimulation, both genotypes robustly upregulated TNF‐α and IL‐6 expression. IFN‐γ levels in WT BMDMs remained low and unchanged between unstimulated and stimulated conditions. In contrast, Znfx1^mut^ BMDMs displayed high basal IFN‐γ expression that decreased modestly upon stimulation but remained significantly elevated compared with stimulated WT BMDMs.

Together, these findings indicate that Znfx1^mut^ BMDMs (i) exhibit heightened pro‐inflammatory cytokine production in the absence of external stimulation and (ii) possess an increased basal propensity for activation. This dysregulated cytokine response in Znfx1^mut^ BMDMs might reflect an underlying intrinsic defect that may contribute to the exaggerated inflammatory phenotype observed in vivo following viral infection.

## 3 Discussion

Our present results showed that LCMV‐infected Znfx1^mut^ mice developed transient features of HLH‐like immune dysregulation while retaining the ability to clear viral infection efficiently. Upon infection, Znfx1^mut^ mice presented with splenomegaly, a low hemoglobin level, hyperferritinemia, and an elevated sIL2R⍺ level. Additional abnormalities resembling those reported in ZNFX1‐deficient patients included elevated serum ALT level and leukocytosis—the latter being uncommon in classical HLH, but documented in a subset of human ZNFX1‐deficient patients [9, 10, 11, 24]. The immune dysregulation observed in Znfx1^mut^ mice was characterized by enhanced T cell expansion and Th1‐skewed differentiation, altered monocyte subset distribution, increased macrophage infiltration into the liver, and elevated baseline cytokine production in macrophages in vitro. Together, these findings support an essential role for ZNFX1 in maintaining immune homeostasis during viral infection and establish Znfx1^mut^ mice as a relevant model for studying virally induced immune dysregulation in ZNFX1 deficiency.

A notable difference between our model and the clinical phenotype of ZNFX1‐deficient patients was the relatively mild and self‐limited nature of the observed inflammation in infected Znfx1^mut^ mice. Many patients with ZNFX1 deficiency present with severe HLH‐like episodes that require immunosuppressive therapy [9, 10, 11, 24]. In contrast, LCMV‐infected Znfx1^mut^ mice developed nonsevere inflammatory manifestations that resolved spontaneously. The relatively low LCMV dose (200 PFU) and the use of a LCMV strain causing acute infection, combined with the efficient viral clearance by Znfx1^mut^ mice, may have limited the duration of antigenic stimulation required to drive more severe HLH‐like pathology. As a result, immune parameters peaked at different time points post infection, and differences in serum cytokine levels, early T cell activation markers, and monocyte counts may have been missed by the time points analyzed in this study.

Our findings contrast with those of Wang et al. [12], who reported minimal systemic inflammation in a distinct *Znfx1* knockout mouse strain under viral challenge. Several methodological differences may explain this divergence. Their study employed vesicular stomatitis virus (VSV), whereas we used LCMV—a virus with a well‐established capacity to reveal immune regulatory defects, particularly in monogenic HLH models. Moreover, differences in CRISPR targeting strategies, genetic backgrounds, viral kinetics, and the specific immunological readouts assessed likely contributed to the distinct outcomes. Importantly, our study focused directly on HLH‐related features previously documented in human patients [9, 10, 11], and is the first murine model system to mimic clinical aspects of multisystem inflammation in ZNFX1 deficiency.

The HLH‐like pathology observed in infected Znfx1^mut^ mice was accompanied by dysregulated T cell, monocyte, and macrophage responses. Upon infection, Znfx1^mut^ mice developed selective proliferation of Th1‐skewed effector memory CD4^+^ T cells, which are key drivers of IFN‐γ‐dominated inflammatory responses [25, 27, 28]. The absence of differences in OX40, CD69, CD27, CD39, or terminal differentiation markers suggests that the T cell dysregulation reflects a qualitative shift in differentiation rather than a global increase in activation. Whether this phenotype is driven by intrinsic T cell abnormalities or by altered extrinsic cues remains unclear. An intrinsic defect would be expected to entail changes in activation; however, the transient phenotype may have limited detectability. Alternatively, extrinsic factors—such as an altered cytokine milieu, differences in antigen‐presenting cell function, or changes in tissue recruitment—may preferentially promote Th1‐skewing in Znfx1^mut^ mice [29]. CD8^+^ T cells, while phenotypically similar in many respects, nonetheless expressed more CXCR3, suggesting that they too experienced altered environmental cues. Taken together, these findings suggest a dysregulated T cell response, although more experiments will be required to fully elucidate the underlying causes.

Alterations were also evident in the myeloid compartment. Infected Znfx1^mut^ mice showed a higher proportion of Ly6C^int^ and a lower proportion of Ly6C^high^ monocytes. Ly6C^high^ monocytes are classical inflammatory monocytes capable of secreting pro‐inflammatory cytokines and extravasating into inflamed tissues [30, 31, 32]. In contrast, nonclassical Ly6C^low^ monocytes are associated with vascular patrolling and tissue repair [33, 34]. Ly6C^int^ monocytes are heterogeneous and exhibit features of both Ly6C^low^ and Ly6C^high^ subsets [35, 36]. In view of the relative shift toward Ly6C^int^ monocytes in the blood and spleen, it is possible that the infection of Znfx1^mut^ mice with LCMV resulted in more extravasation of Ly6C^high^ monocytes into tissues. This extravasation might have resulted in greater macrophage infiltration into the liver. Overall, the larger area occupied by macrophages in the livers of infected Znfx1^mut^ mice and the presence of hemophagocytosis further support heightened macrophage activation.

Quantitative in vitro imaging assays of TNF‐⍺, IL‐6, and IFN‐γ production by BMDMs provided additional insight into macrophage dysregulation. Znfx1^mut^ BMDMs produced more pro‐inflammatory cytokines under unstimulated conditions, whereas WT BMDMs required poly(I:C) stimulation to produce comparable cytokine levels. This intrinsic heightened responsiveness was not present in monocytes before differentiation, suggesting that the defect may emerge during macrophage maturation or as a consequence of altered responses to M‐CSF. Although in vitro systems do not fully mirror the behavior of macrophages in vivo, these findings identify ZNFX1 as an important regulator of macrophage cytokine control. The absence of elevated systemic cytokines in infected Znfx1^mut^ mice may reflect time‐dependent cytokine kinetics—systemic cytokines can rise and fall within hours [37, 38]—or the possibility that dysregulated cytokine production remains localized within tissues. Although an exploration of the molecular defects driving inflammation in the absence of ZNFX1 was outside the scope of the present study, these results might suggest that the immune dysregulation in ZNFX1 deficiency results from intrinsic defects in macrophage cytokine control.

In conclusion, our work demonstrates that LCMV‐infected Znfx1^mut^ mice recapitulate several clinical and immunological features of ZNFX1 deficiency, including aspects of HLH‐like inflammation. This model provides a valuable foundation for dissecting the immunopathogenesis of ZNFX1 deficiency and can be applied to future mechanistic and therapeutic studies.

## 4 Data Limitations and Perspectives

Mechanistic dissection of how ZNFX1 regulates immune signaling and contributes to HLH pathogenesis was beyond the scope of this study, which focused on establishing and characterizing an in vivo model of ZNFX1 deficiency. Future work will be required to determine whether impaired viral sensing, infected target cell killing, type I IFN signaling, or inflammasome signaling directly contributed to the aberrant inflammation observed in Znfx1^mut^ mice. The LCMV‐based model developed here now provides a controlled experimental system in which to test targeted immunomodulatory interventions, including JAK inhibition, IFN‐γ blockade, inflammasome inhibition, IFNAR1 blockade, and other emerging therapeutic approaches. These studies may yield insights into the molecular pathways driving inflammation in ZNFX1 deficiency and could inform clinical strategies for managing this rare but severe disorder.

## 5 Material and Methods

### 5.1 Mice

C57BL/6J and C57BL/6NJ‐*Znfx1^em1(IMPC)J^/*Mmjax mice were obtained from Jackson Laboratories. Mice were housed in specific pathogen‐free conditions at the University of Zurich (Zurich, Switzerland). All studies were approved by the Zurich Cantonal Veterinary Office and conducted in accordance with the regulations of the University of Zurich's Laboratory Animal Service Center.

### 5.2 BMDM Cultures

Eight‐ to twelve‐week‐old mice were euthanized and sprayed with 70% ethanol. The femurs and tibias were removed, cleaned (to remove tissue remnants), and transferred to a Falcon tube containing Dulbecco's PBS (DPBS, Gibco) on ice. The bones were cut at one end and placed in a 0.5 mL sterile Eppendorf tube that had previously been punctured with an 18G needle and nested in a 1.5 mL sterile Eppendorf tube. The bone marrow was transferred into the 1.5 mL Eppendorf tube by centrifugation at 4000×*g* for 15 s. Red Blood Cell Lysis Buffer (Sigma‐Aldrich) was added and incubated for 10 min at room temperature. The cells were cultured in Iscove's modified Dulbecco's medium (IMDM, Gibco) supplemented with 1% GlutaMax (Gibco), 1% Pen Strep 5000 U/mL (Gibco), 10% fetal bovine serum (Sigma‐Aldrich), and 40 ng/mL M‐CSF (Miltenyi) for 6–9 days, until they developed the characteristic appearance of macrophages. The cells were detached with TrypLE Express Enzyme (Gibco), counted, and frozen in 90% fetal bovine serum (Sigma‐Aldrich) with 10% DMSO (Sigma‐Aldrich).

### 5.3 Sanger Sequencing of Murine Samples

BMDMs were stimulated with 500 ng/mL Poly(I:C) HMW Lyovec (Invitrogen) for 18 h. RNA was extracted with TRIzol (Invitrogen) and purified with the PureLink RNA Mini Kit (Fisher Scientific). Iscript Reverse Transcription Supermix (Bio‐Rad) was used to generate cDNA, which was PCR‐amplified using the primers “JAX PCR fwd” (5’‐ATGAGGAAGTCTCTGAGAGGTG‐3’) and “JAX PCR rev” (5’‐ATTCAAGAATCATGGAGTGC‐3’) supplied by Microsynth AG. The primers “JAX Seq fwd” (5’‐TATCATTCAAGGACCTCCTG – 3’) and “JAX Seq rev” (5‐CAGAGATGTACTTCTCCAGGTG‐3’) were used for Sanger sequencing, which was performed by Microsynth AG. Benchling software was used to align the results to the reference sequence.

### 5.4 The LCMV Infection Model and Sample Collection

Eight‐ to ten‐week‐old mice were used for experiments and were randomly assigned to the experimental group or the control group. 200 plaque‐forming units (PFU) of LCMV‐WE (kindly provided by P. Aichele at the University of Freiburg Medical Center (Freiburg, Germany)) or 0.9% saline solution were injected intraperitoneally. The injection volume was 100 µL. Mice were euthanized 7, 9, or 15 days postinfection by CO_2_ asphyxiation. Immediately after euthanasia, the chest cavity was opened, and blood was drawn from the heart into a syringe precoated with heparin (Heparin‐Na 25000 I.E., B. Braun). 50 to 250 µL of whole blood were transferred to a blood collection tube containing potassium EDTA (Microtainer K2E, BD) for whole blood cell differential or flow cytometry analysis and stored at 4°C. The rest of the blood sample was transferred to a standard Eppendorf tube and centrifuged for 10 min at 1200×g. The serum was carefully collected and stored at −80°C. Next, the spleen was removed and weighed, and a small part (around a fifth) was cut off and transferred to an Eppendorf tube containing 1 mL of TRIzol (Invitrogen). The tube was then stored at −80°C. The rest of the spleen was transferred to a Falcon tube containing 1 mL of DPBS (Gibco) and stored at 4°C. The liver was removed and stored in 4% buffered formaldehyde (Sigma‐Aldrich).

### 5.5 RT‐qPCR Assay of the LCMV Load

The spleen sample stored in TRIzol was thawed on ice. A pestle (VWR) was used to break up the tissue, and 200 µL of chloroform (Fisher Scientific) was added. The solution was vortexed and centrifuged at 12000×*g* for 20 min. The aqueous phase containing the RNA was carefully removed, and the RNA was extracted using the PureLink RNA Mini Kit (Fisher Scientific). Iscript Reverse Transcription Supermix (Bio‐Rad) was used to generate cDNA. RT‐q PCR was performed with FastStart Universal SYBR Green Master (Sigma‐Aldrich) with the Bio‐Rad CFX Maestro C1000 Touch Thermo cycler and the following primers: “LCMV fwd” (5’‐TCT CAT CCC AAC CAT TTG CA‐3’), “LCMV rev” (5’‐GGG AAA TTT GAC AGC ACA ACA A‐3’), “β‐Actin fwd” (5’‐CCA GGA GAT GTG GAT CAG CA‐3’), and “β‐Actin rev” (5’‐CTT GCG GTG CAC GAT GG‐3’), ordered from Microsynth AG. The results were analyzed using Bio‐Rad CFX Maestro software.

### 5.6 Whole Blood and Serum Analysis

Whole blood samples were analyzed in the Veterinary Laboratory at the University Animal Hospital Zurich (Zurich, Switzerland). This included whole blood differential analysis and quantification of the hemoglobin level. The serum samples were analyzed using the following kits and according to the manufacturers’ instructions: Mouse Ferritin ELISA kit (Crystal Chem), mouse alanine aminotransferase (ALT) ELISA kit (Abcam), Mouse CD25/IL‐2 R alpha DuoSet ELISA (R&D Systems), and the Legendplex mouse antivirus response panel (13‐plex) (BioLegend). The samples were run in duplicates. The ELISA experiments were analyzed using a BioTek Cytation5 imaging reader. The Legendplex plates were analyzed with a Cytek Aurora Spectral flow cytometer.

### 5.7 Liver Histology and Immunohistochemistry

Liver samples were embedded in paraffin by Sophistolab. H&E staining and anti‐cleaved caspase 3 immunohistochemical staining were performed at the University Hospital Zurich's Department of Pathology and Molecular Pathology. Anti‐F4/80 and anti‐CLEC4F immunohistochemistry staining was performed by Sophistolab. Machine learning algorithms (provided by ilastik) were used for pixel classification of macrophages, vessels, and background tissue cells. The pixel probability maps were exported and used for object classification to improve the distinction between vessels and background tissue. Next, the pixel probability maps and the object classification probability maps were analyzed with Fiji software. Thresholding was performed, and only pixels with at least a 75% chance of belonging to a macrophage or at least a 70% chance of belonging to a vessel were considered, as these probability maps best represented the raw data. The pixel classification masks showing macrophages and the object classification masks depicting vessels were overlaid on the original image, using Fiji software. Fiji was also used to calculate the macrophage‐positive area and the vessel‐positive area in each image. Lastly, the macrophage‐positive area was expressed as a percentage of the total tissue area, in order to account for variations in vessel number and size in each image.

### 5.8 Flow Cytometry of Blood Cells and Splenocytes

To create a single‐cell suspension, the spleens were mechanically disaggregated using a 70 µM cell strainer (Corning). Red blood cell lysis buffer (Sigma‐Aldrich) was added. Cells were stained with Zombie Aqua (BioLegend) in PBS for 20 min at room temperature. Surface staining was performed in DPBS (Gibco) with 5% fetal bovine serum (Sigma‐Aldrich) and 0.1% sodium azide (Santa Cruz Biotechnology) for 30 min at 4°C. For intracellular staining, the cells were subsequently treated with the eBioscience Foxp3 Transcription Factor Staining kit (Thermo Scientific), according to the manufacturer's instructions. The data were acquired with a Cytek Aurora Spectral flow cytometer and analyzed with SpectroFlo software (Cytek). The gating strategy is shown in Figure S5. Splenocytes not used for flow cytometry were frozen in 90% fetal bovine serum (Sigma‐Aldrich) and 10% DMSO (Sigma‐Aldrich).

### 5.9 In Vitro T Cell Stimulation

Splenocytes harvested 9 days post‐LCMV infection were thawed on ice and resuspended in RPMI 1640 medium (Thermo Scientific) supplemented with 1% GlutaMax Supplement (Gibco), 1% Pen Strep 5000 U/mL (Gibco), and 10% fetal bovine serum (Sigma‐Aldrich). The cells were stimulated with 10 µM gp33‐41 (Med Chem Express), 10 µM gp61‐80 (Med Chem Express), or 0.08 µM phorbol 12‐myristate 13‐acetate (PMA), and 1.34 µM ionomycin (eBioscience Cell Stimulation Cocktail, Thermo Scientific). The stimulation lasted for 5 h in the presence of the intracellular protein transport inhibitor brefeldin A (Thermo Scientific). Surface staining was performed as described for the flow cytometry of splenocytes. For intracellular staining, the cells were subsequently treated with the BD Cytofix/Cytoperm kit, according to the manufacturer's instructions. The data were acquired with a Cytek Aurora Spectral flow cytometer and analyzed with SpectroFlo software (Cytek). The gating strategy is shown in Figure S5.

### 5.10 In Vitro Monocyte Stimulation

Bone marrow cells were harvested as described in section BMDM culture and cultured in RPMI 1640 medium (Thermo Scientific) supplemented with 1% GlutaMax Supplement (Gibco), 1% Pen Strep 5000 U/mL (Gibco), and 10% fetal bovine serum (Sigma‐Aldrich) overnight. Cells were stimulated with 2 µg/mL ssRNA40/Lyovec (InvivoGen) for a total of 8 h. BD GolgiPlug protein transport inhibitor (BD) was added to all wells for a total of 6 h. Cells were detached by pipetting and processed for flow cytometry as described in the section flow cytometry of blood cells and splenocytes. For intracellular staining, the BD Cytofix/Cytoperm kit was used, according to the manufacturer's instructions.

### 5.11 Antibodies for Flow Cytometry

Zombie Aqua (BioLegend), CD45 BB515 (Clone 30 F11, BD), CD3 Alexa Fluor 700 (Clone 17A2, BioLegend), CD19 PE‐Cy7 (Clone 6D5, BioLegend), NK‐1.1 BV605 (Clone PK136, BioLegend), CD11c PE‐Cy5 (Clone N418, BioLegend), I‐A/I‐E BV785 (Clone M5/114.15.2, BioLegend), CD11b BUV395 (Clone M1/70, BD), Ly‐6G BV711 (Clone 1A8, BioLegend), Ly‐6C BV570 (Clone HK1.4, BioLegend), CD8a APC‐Cy7 (Clone 53–6.7, BioLegend), CD4 PE‐Texas Red (Clone RM4‐5, Thermo Scientific), CD62L Pacific Blue (Clone MEL‐14, BioLegend), CD44 BUV737 (Clone IM7, BD), CD183 BUV661 (Clone CXCR3‐173, BD), CD366 BV711 (Clone RMT3‐23, BioLegend), IFN‐γ PE‐Alexa Fluor 610 (Clone XMG1.2, Thermo Scientific), Ki‐67 Alexa Fluor 532 (Clone SolA15, Thermo Scientific), T‐bet BV785 (Clone 4B10, BioLegend), CD134 BV605 (Clone OX‐86, BioLegend), CD69 BV785 (Clone H1.2F3, BioLegend), CD27 BV650 (Clone LG.3A10, BioLegend), CD39 PE‐Cy5 (Clone Duha59, BioLegend), CD279 eFluor 450 (Clone J43, Thermo Scientific), Tox Alexa Fluor 647 (Clone NAN448B, BD), Granzyme B PE‐Cy5.5 (Clone NGZB, Thermo Scientific), CX3CR1 Alexa Fluor 647 (Clone SA011F11, BioLegend), CD192 BV421 (Clone SA203G11, BioLegend), F4/80 BUV496 (Clone T45‐2342, BD).

### 5.12 Microscopy Assay of Intracellular Cytokine Production by BMDMs

A frozen vial of BMDMs was thawed and diluted in IMDM (Gibco) supplemented with 1% GlutaMax Supplement (Gibco), 1% Pen Strep 5000 U/mL (Gibco), 10% fetal bovine serum (Sigma‐Aldrich), and 40 ng/mL M‐CSF (Miltenyi). Live cells were counted using 0.4% Trypan Blue solution (Gibco). A total of 50,000 live cells were added to each well of a microscopy plate. Once the cells had attached, the medium was replaced with fresh medium; this removed dead cells and traces of DMSO (Sigma‐Aldrich) left over from the freezing medium. After 2 days in culture, the medium was changed again. Fresh medium was added to the control wells. To stimulate cytokine production, medium with 2 µg/mL Poly(I:C) HMW (InvivoGen) was added for a total of 8 h. BD GolgiPlug protein transport inhibitor (BD) was added to all wells for a total of 6 h. Cells were fixed with prewarmed paraformaldehyde (Electron Microscopy Sciences, end concentration: 4%) for 30 min at room temperature. 1 mg/mL sodium borohydride (Sigma‐Aldrich) in PBS was added to quench the autofluorescence, with shaking for 10 min at room temperature three times in total. 0.5% Triton X‐100 (Sigma‐Aldrich) in PBS was added to permeabilize the cells, and the sample was shaken for 15 min at room temperature. Intercept Blocking Buffer (Chemie Brunschwig AG) was added for 1 h with shaking at room temperature. Rat anti‐mouse IL‐6 Alexa Fluor 488 (BD), rat anti‐mouse TNF‐⍺ PE (BioLegend), and rat anti‐mouse IFN‐γ Alexa Fluor 488 (BioLegend) were used to stain an individual well each for 2 h with shaking in the dark at room temperature. Donkey anti‐rat Alexa Fluor 568 (Thermo Scientific) was added as a secondary stain to wells previously stained for IFN‐γ or IL‐6, with shaking for 1 h at room temperature in the dark. Pacific Blue Succinimidyl Ester (Thermo Scientific) was resuspended in DMSO to 10 mg/mL (Sigma‐Aldrich) and diluted 1:20,000 in PBS with 0.1 M sodium bicarbonate (Sigma‐Aldrich) and 0.0025 M sodium carbonate (Sigma‐Aldrich). The diluted Pacific Blue Succinimidyl Ester was added to the wells, and the sample was shaken for 15 min at room temperature in the dark. The wells were washed three times with PBS and then imaged in imaging buffer with 0.7 M N‐acetyl‐L‐cysteine (Sigma‐Aldrich) and 200 mM HEPES (Thermo Scientific) in water (adjusted to pH7.4 with sodium hydroxide (Sigma‐Aldrich)), using a microscope (Operetta, Perkin Elmer).

For quantitative assays, we measured single‐cell cytokine expression distributions in each mouse and subsequently grouped the data from three mice per condition. To account for interindividual variability in cell counts, we normalized the data accordingly. Statistical comparisons were performed by modeling the normalized single‐cell data as a three‐component Gaussian mixture model (GMM). This approach enabled us to capture the heterogeneity in cytokine expression and derive key variables (including the weighted mean, covariance, and component proportions), which were then used for the intergroup comparisons (Tables S1, S2, and S3).

### 5.13 Statistics

The statistical tests used are specified in the figure legends. The data from the in vitro BMDM macrophage experiment and the corresponding statistical tests were visualized with JupyterLab software. All other graphic and statistical analyses were performed with GraphPad Prism software (version 10.1.0).

## Author Contributions

Conceptualization and methodology: Diana Tintor, Samantha Milanesi, Ola Sabet, and Jana Pachlopnik Schmid. Investigation – animal experiments: Diana Tintor, Samantha Milanesi, Tommaso Marchetti, Junyi Chen, Severin Walser, and Julius Köppen. Investigation – other experiments: Diana Tintor, Samantha Milanesi, Ola Sabet, Achim Weber, and Tiziana Lorenzini. Data visualization: Diana Tintor and Ola Sabet. Funding acquisition: Jana Pachlopnik Schmid. Project administration and supervision: Jana Pachlopnik Schmid. Writing – original draft: Diana Tintor, Ola Sabet, Achim Weber, and Jana Pachlopnik Schmid. Writing – review and editing: All authors contributed to the review and revision of the manuscript and approved the final version.

## Funding

This work was funded by the Swiss National Science Foundation (project reference: 320030_205097, to D.T., S.W., and J.P.S.), the Wolfermann‐Nägeli‐Stiftung (a grant to D.T., S.W., and J.P.S.), and the University of Zurich (the CYTIMM‐Z Clinical Research Priority Program, to T.M. and J.P.S., and the ITINERARE, a University Research Priority Program to J.C. and J.P.S.

## Conflicts of Interest

The authors declare no conflicts of interest.

## Ethics Approval Statement

Experiments on mice were approved by the Zurich Cantonal Veterinary Office, under the terms of licenses ZH158/2020 and ZH008/2023.

## Supporting information




**Supporting File**: eji70141‐sup‐0001‐SupMat.pdf.

## Data Availability

The raw data that support the findings of this study are available upon request from the corresponding author.
