# Supplementary material for: A Novel Murine Model of Hemophagocytic Lymphohistiocytosis‐Like Inflammation in ZNFX1 Deficiency
Source: Eur J Immunol. 2026 Feb 4;56(2):e70141. doi: 10.1002/eji.70141 (PMC12869469; doi:10.1002/eji.70141)
Supplement: Supplementary file 1 — Supporting File: eji70141‐sup‐0001‐SupMat.pdf. [file EJI-56-e70141-s001.pdf]

Supplementary material to:

A novel murine model of hemophagocytic lymphohistiocytosis-like inflammation in ZNFX1 deficiency

Authors: Diana Tintor[1,2], Samantha Milanesi[1,2], Tommaso Marchetti[1,2], Tiziana Lorenzini[1,2], Severin Walser[1,2], Junyi Chen[1,2], Julius Köppen[1,2], Achim Weber[4,5], Ola Sabet[1,2,3] and Jana Pachlopnik Schmid[1,2]

Affiliations:
[1]Division of Immunology and University Children's Hospital Research Center, University Children's Hospital Zurich, Zurich, Switzerland.
[2]Pediatric Immunology, Faculty of Medicine, University of Zurich, Zurich, Switzerland.
[3]Children's Cancer Hospital Egypt 57357, Cairo, Egypt.
[4]Department of Pathology and Molecular Pathology, University Hospital Zurich and University of Zurich, Zurich, Switzerland.
[5]Institute of Molecular Cancer Research (IMCR), University of Zurich, Zurich, Switzerland.

Corresponding Author: jana.pachlopnik@kispi.uzh.ch

Supplementary Figures:

Supplementary Figure 1:

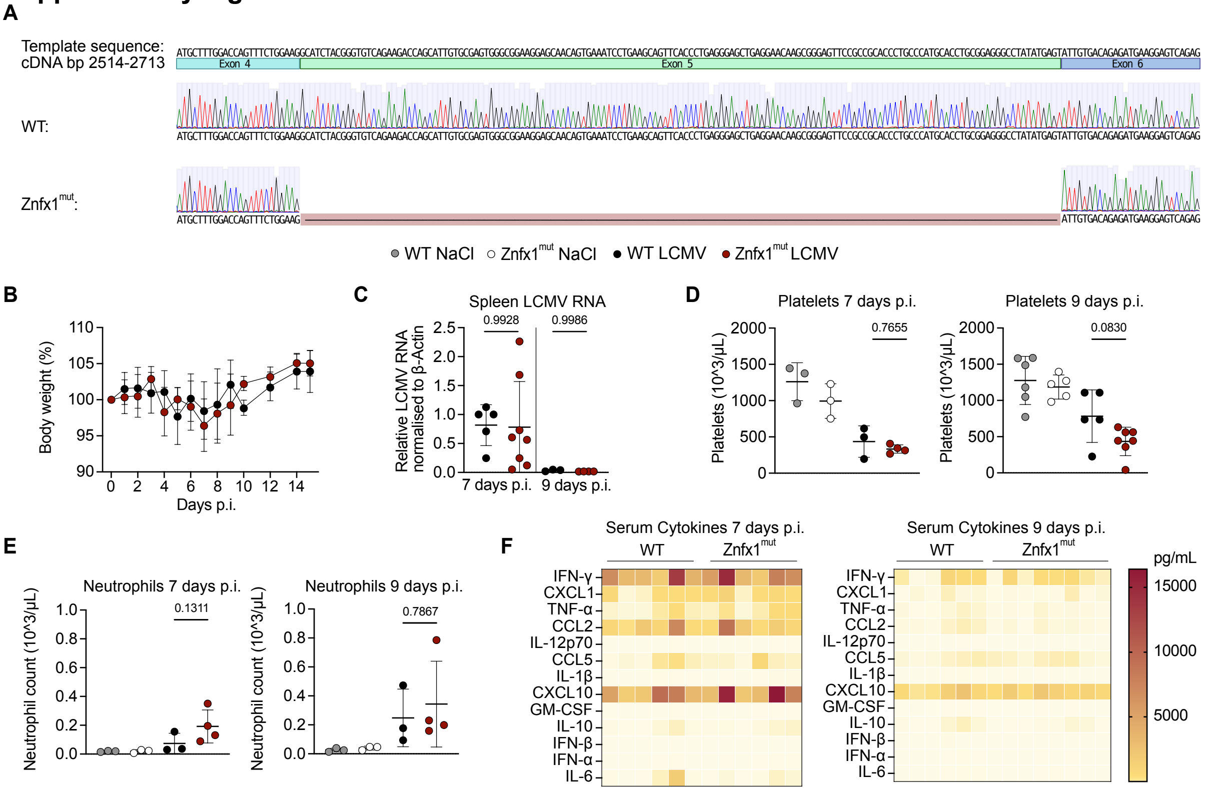

Fig. S1: LCMV-infected Znfx1^{mut} mice develop transient, HLH-like inflammation that mimics clinical aspects of patients with ZNFX1 deficiency.
(A) Sanger Sequencing results from the PCR product obtained after PCR amplification of extracted mRNA from bone marrow-derived macrophages previously stimulated with 500 ng/mL Poly(I:C) Lyovec

for 18 hours. Top row: Excerpt of the Znfx1 cDNA template sequence spanning the base pairs (bp) 2514-2713. Middle row: Sequence measured for the PCR product from WT mice. Bottom row: Sequence measured for the PCR product from Znfx1^{mut} mice. **(B)** Mouse weight as a percentage of starting body weight over the course of 15 days post-infection (p.i.) with 200 PFU of LCMV strain WE. **(C)** Relative LCMV RNA expression normalized to RNA from the housekeeping gene β-Actin. The RNA was extracted from spleens 7 days (left panel) and 9 days (right panel) p.i. with 200 PFU of LCMV strain WE. **(D)** Platelet count in whole blood 7 days (left panel) and 9 days (right panel) p.i. with 200 PFU of LCMV strain WE. **(E)** Neutrophil count in whole blood 7 days (left panel) and 9 days (right panel) p.i. with 200 PFU LCMV strain WE. **(F)** Serum cytokines measured by multiplexed flow cytometry 7 days (left panel) and 9 days (right panel) p.i. with 200 PFU LCMV strain WE. For all plots, the data were analyzed in a one-way analysis of variance with Šidák's correction for multiple comparisons. The graphs display the mean and the standard deviation. S1B shows pooled results with the sample size ranging from 3 to 21. The data for euthanasia time points 7, 9, and 15 days p.i. were generated in two to four independent experiments. For S1C-E each dot represents an individual mouse and the data were generated in one to three independent experiments. p-values are indicated above the plots.

Supplementary Figure 2:

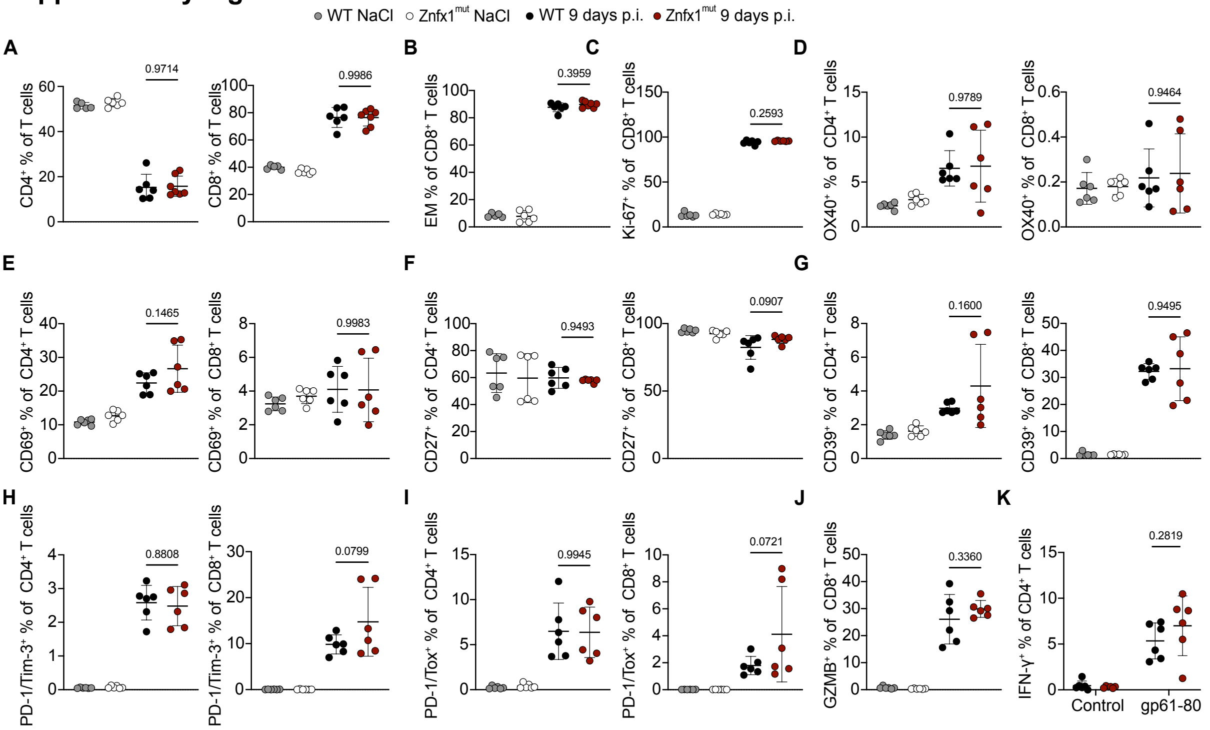

Fig. S2: LCMV-infected Znfx1^{mut} mice show T cell expansion and more pronounced Th1 polarisation.

(A) Flow cytometry plot of CD4 (left panel) and CD8 (right panel) expression on T cells in the spleen. **(B)** Flow cytometry plot of CD62L and CD44 expression on CD8$^+$ T cells in the spleen. EM: effector memory. **(C)** Flow cytometry plot of Ki-67 expression on CD8$^+$ T cells in the spleen. **(D)** Flow cytometry plot of OX-40 expression on CD4$^+$ (left panel) and CD8$^+$ T cells (right panel) in the spleen. **(E)** Flow cytometry plot of CD69 expression on CD4$^+$ (left panel) and CD8$^+$ T cells (right panel) in the spleen. **(F)** Flow cytometry plot of CD27 expression on CD4$^+$ (left panel) and CD8$^+$ T cells (right panel) in the spleen. **(G)** Flow cytometry plot of CD39 expression on CD4$^+$ (left panel) and CD8$^+$ T cells (right panel) in the spleen. **(H)** Flow cytometry plot of PD-1 and Tim-3 co-expression on CD4$^+$ (left panel) and CD8$^+$ T cells (right panel) in the spleen. **(I)** Flow cytometry plot of PD-1 and Tox co-expression on CD4$^+$ (left panel) and CD8$^+$ T cells (right panel) in the spleen. **(J)** Flow cytometry plot of Granzyme B (GZMB) expression in CD8$^+$ T cells taken from the spleen after *in vitro* stimulation with 0.08 μM phorbol 12-myristate 13-acetate, 1.34 μM ionomycin and brefeldin A for 5 hours. **(K)** Flow cytometry plot of IFN-γ expression in CD4$^+$ T cells after *in vitro* stimulation with 10 μM gp61-80 and brefeldin A for 5 hours. All plots show cells from mice mock-infected with NaCl or 9 days post-infection (p.i.) with 200 PFU of LCMV strain WE. The data were analyzed in a one-way analysis of variance with Šidák's correction for multiple comparisons. The graphs display the mean and the standard deviation. Each dot represents an individual mouse, and all plots show the data from at least two independent experiments. p-values are indicated above the plots.

Supplementary Figure 3:

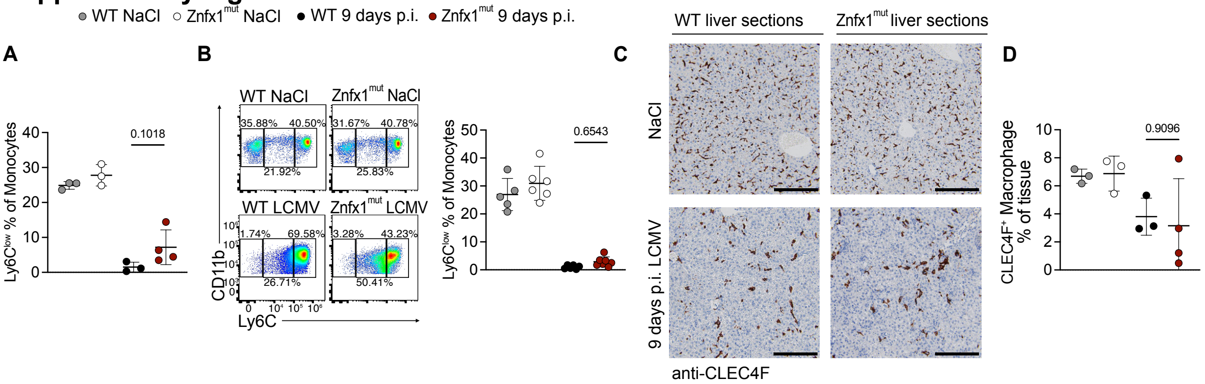

Fig. S3: LCMV-infected Znfx1^{mut} mice show altered Ly6C expression patterns in monocytes and a greater abundance of macrophages in the liver 9 days p.i.

(A) Ly6C^{low} monocytes in whole blood. **(B)** Ly6C^{low} monocytes in the spleen. **(C)** Liver sections immunohistochemically stained with anti-CLEC4F, 20x magnification. Scale bar: 100 µM. **(D)** Quantification of CLEC4F^{+} macrophages in the liver. All plots show cells from mice mock-infected with NaCl or 9 days post-infection (p.i.) with 200 PFU of LCMV strain WE. The data were analyzed in a one-way analysis of variance with Šidák's correction for multiple comparisons. The graphs display the mean and the standard deviation. Each dot represents an individual mouse. The data are from one (S3A, S3C, S3D) or two (S3B) independent experiments. p-values are indicated above the plots.

Supplementary Figure 4:

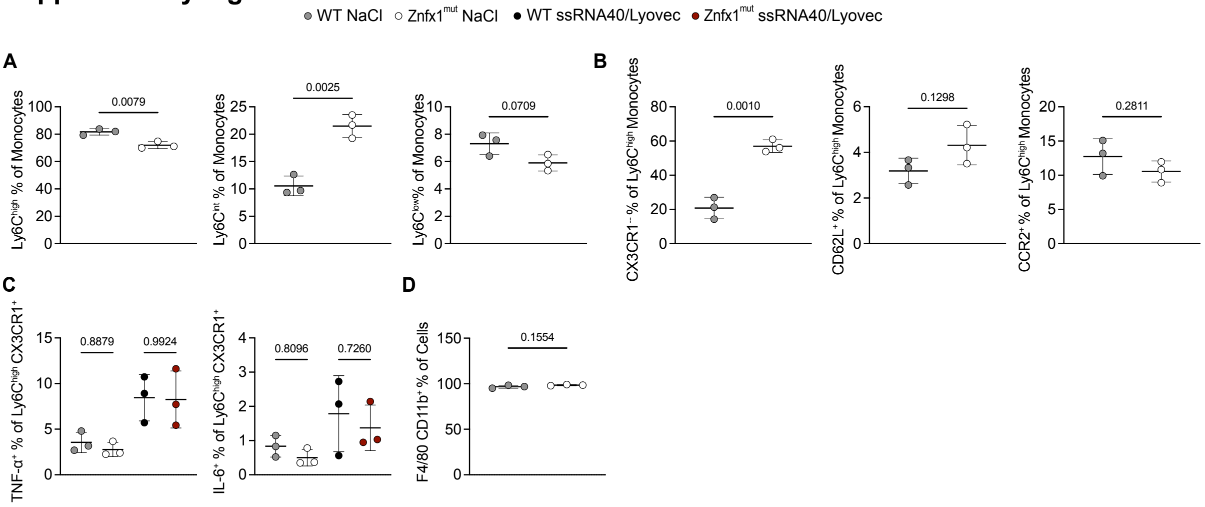

Fig. S4: BMDMs from Znfx1^{mut} mice produce more pro-inflammatory cytokines in an unstimulated state

(A) Ly6C^{high} (left panel), Ly6C^{intermediate} (Ly6C^{int,} middle panel) and Ly6C^{low} monocytes (right panel) in the bone marrow. **(B)** Flow cytometry plot of CX3CR1^{-} (left panel), CD62L^{+} (middle panel), and CCR2^{+} (right panel) Ly6C^{high} monocytes in the bone marrow. **(C)** Flow cytometry plot of TNF-α (left panel) and IL-6 (right panel) expression in Ly6C^{high} CX3CR1^{+} monocytes taken from the bone marrow after stimulation with 2 µg/mL ssRNA40/Lyovec for 8 hours, with brefeldin A for 5 hours. **(D)** Flow cytometry plot of F4/80 and CD11b co-expression on bone marrow cells differentiated into bone marrow-derived macrophages for 7 days with murine colony-stimulating factor. All plots show cells harvested from the bone marrow of mice. The data were analyzed in a one-way analysis of variance with Šidák's correction for multiple comparisons. The graphs display the mean and the standard deviation. Each dot represents an individual mouse. The data are from one independent experiment. p-values are indicated above the plots.

Supplementary Figure 5:

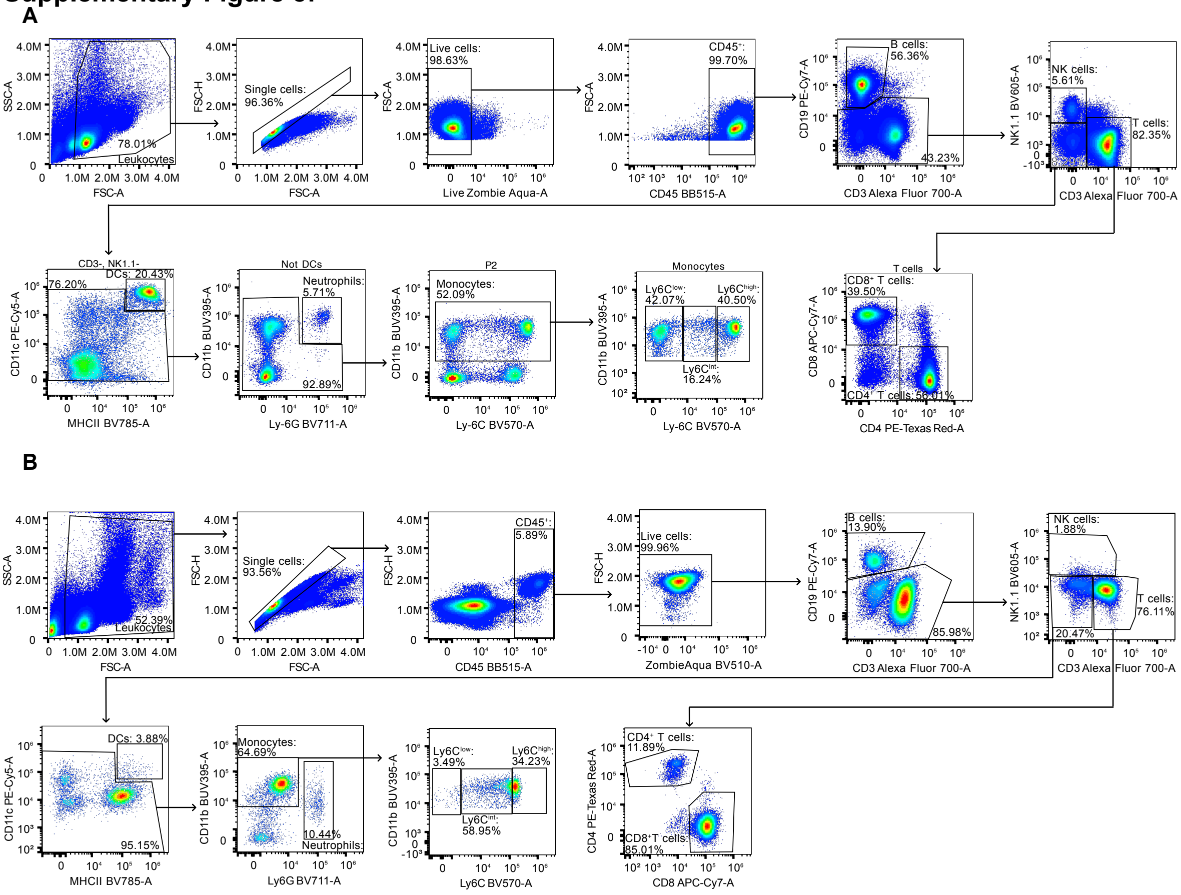

Fig. S4: Flow cytometry gating strategy. (A) Gating strategy for splenocytes. (B) Gating strategy for whole blood.

Supplementary Tables:

Supplementary Table 1: Statistical mean, covariance and weight of each component of the Gaussian mixture models used to describe the log10 TNF-α intensity of BMDMs.

**TNF-α intensity (log10)**		Mean	Covariance	Weight
WT	Component 1	2.46	0.03	0.39
	Component 2	2.62	0.08	0.42
	Component 3	2.73	0.13	0.19
Znfx1^{mut}	Component 1	2.45	0.02	0.24
	Component 2	2.62	0.08	0.55
	Component 3	2.72	0.13	0.20
WT + Poly(I:C) HMW	Component 1	2.45	0.02	0.20
	Component 2	2.63	0.10	0.49
	Component 3	2.73	0.14	0.31
Znfx1^{mut} + Poly(I:C) HMW	Component 1	2.45	0.02	0.30
	Component 2	2.66	0.10	0.62
	Component 3	2.82	0.23	0.08

Supplementary Table 2: Statistical mean, covariance and weight of each component of the Gaussian mixture models used to describe the log10 IL-6 intensity of BMDMs.

**IL-6 intensity (log10)**		Mean	Covariance	Weight
WT	Component 1	2.62	0.05	0.34
	Component 2	2.71	0.05	0.47
	Component 3	2.85	0.09	0.19
Znfx1^{mut}	Component 1	2.66	0.06	0.54
	Component 2	2.82	0.08	0.28
	Component 3	3.03	0.13	0.18
WT + Poly(I:C) HMW	Component 1	2.64	0.07	0.46
	Component 2	2.87	0.10	0.36
	Component 3	3.12	0.14	0.18
Znfx1^{mut} + Poly(I:C) HMW	Component 1	2.65	0.07	0.48
	Component 2	2.86	0.10	0.32
	Component 3	3.10	0.15	0.20

Supplementary Table 3: Statistical mean, covariance and weight of each component of the Gaussian mixture models used to describe the log10 IFN-γ intensity of BMDMs.

**IFN-γ intensity (log10)**		Mean	Covariance	Weight
WT	Component 1	2.63	0.04	0.50
	Component 2	2.75	0.06	0.33
	Component 3	2.91	0.10	0.17
Znfx1^{mut}	Component 1	2.63	0.06	0.38
	Component 2	2.82	0.08	0.38
	Component 3	3.00	0.11	0.24
WT + Poly(I:C) HMW	Component 1	2.62	0.05	0.54
	Component 2	2.77	0.07	0.33
	Component 3	2.95	0.11	0.13
Znfx1^{mut} + Poly(I:C) HMW	Component 1	2.62	0.05	0.45
	Component 2	2.78	0.08	0.35
	Component 3	3.02	0.13	0.20
